# VDGE: a data repository of variation database for gene-edited animals across multiple species

**DOI:** 10.1093/nar/gkae956

**Published:** 2024-10-29

**Authors:** Wenwen Shi, Enhui Jin, Lu Fang, Yanling Sun, Zhuojing Fan, Junwei Zhu, Chengzhi Liang, Ya-Ping Zhang, Yong Q Zhang, Guo-Dong Wang, Wenming Zhao

**Affiliations:** State Key Laboratory of Molecular and Developmental Biology, Institute of Genetics and Developmental Biology, Chinese Academy of Sciences, No. 1 West Beichen Road, Chaoyang District, Beijing 100101, China; National Genomics Data Center, China National Center for Bioinformation, No. 1 West Beichen Road, Chaoyang District, Beijing 100101, China; Beijing Institute of Genomics, Chinese Academy of Sciences, No. 1 West Beichen Road, Chaoyang District, Beijing 100101, China; University of Chinese Academy of Sciences, No.1 Yanqihu East Rd, Huairou District, Beijing 101408, China; Key Laboratory of Seed Innovation, Institute of Genetics and Developmental Biology, Chinese Academy of Sciences, No. 1 West Beichen Road, Chaoyang District, Beijing 100101, China; National Genomics Data Center, China National Center for Bioinformation, No. 1 West Beichen Road, Chaoyang District, Beijing 100101, China; Beijing Institute of Genomics, Chinese Academy of Sciences, No. 1 West Beichen Road, Chaoyang District, Beijing 100101, China; Lester and Sue Smith Breast Center, Baylor College of Medicine, One Baylor Plaza, Cambridge Street, Houston, TX 77030, USA; Department of Molecular and Human Genetics, Baylor College of Medicine, One Baylor Plaza, Cambridge Street, Houston, TX 77030, USA; National Genomics Data Center, China National Center for Bioinformation, No. 1 West Beichen Road, Chaoyang District, Beijing 100101, China; Beijing Institute of Genomics, Chinese Academy of Sciences, No. 1 West Beichen Road, Chaoyang District, Beijing 100101, China; National Genomics Data Center, China National Center for Bioinformation, No. 1 West Beichen Road, Chaoyang District, Beijing 100101, China; Beijing Institute of Genomics, Chinese Academy of Sciences, No. 1 West Beichen Road, Chaoyang District, Beijing 100101, China; University of Chinese Academy of Sciences, No.1 Yanqihu East Rd, Huairou District, Beijing 101408, China; Key Laboratory of Seed Innovation, Institute of Genetics and Developmental Biology, Chinese Academy of Sciences, No. 1 West Beichen Road, Chaoyang District, Beijing 100101, China; Key Laboratory of Genetic Evolution and Animal Models, Yunnan Key Laboratory of Molecular Biology of Domestic Animals, Kunming Institute of Zoology, Chinese Academy of Sciences, No.17 Longxin Road, Panlong District, Kunming 650201, China; State Key Laboratory of Molecular and Developmental Biology, Institute of Genetics and Developmental Biology, Chinese Academy of Sciences, No. 1 West Beichen Road, Chaoyang District, Beijing 100101, China; University of Chinese Academy of Sciences, No.1 Yanqihu East Rd, Huairou District, Beijing 101408, China; School of Life Sciences, Hubei University, 368 Youyi Avenue, Wuchang District, Wuhan 430062, China; University of Chinese Academy of Sciences, No.1 Yanqihu East Rd, Huairou District, Beijing 101408, China; Key Laboratory of Genetic Evolution and Animal Models, Yunnan Key Laboratory of Molecular Biology of Domestic Animals, Kunming Institute of Zoology, Chinese Academy of Sciences, No.17 Longxin Road, Panlong District, Kunming 650201, China; National Genomics Data Center, China National Center for Bioinformation, No. 1 West Beichen Road, Chaoyang District, Beijing 100101, China; Beijing Institute of Genomics, Chinese Academy of Sciences, No. 1 West Beichen Road, Chaoyang District, Beijing 100101, China; University of Chinese Academy of Sciences, No.1 Yanqihu East Rd, Huairou District, Beijing 101408, China

## Abstract

Gene-edited animals are crucial for addressing fundamental questions in biology and medicine and hold promise for practical applications. In light of the rapid advancement of gene editing technologies over the past decade, a dramatically increased number of gene-edited animals have been generated. Genome editing at off-target sites can, however, introduce genomic variations, potentially leading to unintended functional consequences in these animals. So, there is an urgent need to systematically collect and collate these variations in gene-edited animals to aid data mining and integrative in-depth analyses. However, existing databases are currently insufficient to meet this need. Here, we present the Variation Database of Gene-Edited animals (VDGE, https://ngdc.cncb.ac.cn/vdge), the first open-access repository to present genomic variations and annotations in gene-edited animals, with a particular focus on larger animals such as monkeys. At present, VDGE houses 151 on-target mutations from 210 samples, and 115,710 variations identified from 107 gene-edited and wild-type animal trios through unified and standardized analysis and concurrently provides comprehensive annotation details for each variation, thus facilitating the assessment of their functional consequences and promoting mechanistic studies and practical applications for gene-edited animals.

## Graphical Abstract

**Graphical Abstract:**
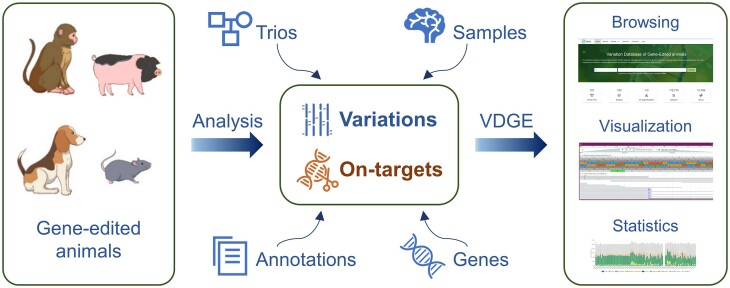

## Introduction

With the rapid advancement of the clustered regularly interspaced short palindromic repeat (CRISPR) gene editing technology (1,2), a dramatically increased number of gene-edited animals have been generated over the past decade. Large gene-edited animals such as monkeys, pigs and dogs are utilized increasingly in studies of human disease modelling, regenerative medicine and agricultural breeding (3–9). Gene editors can induce either DNA double-stranded breaks (DSBs) (2,10) or single-stranded breaks (SSBs) (11–14) at on- or off-target sites, potentially leading to genomic variations in gene-edited animals that differ from those in their parents and in non-gene-edited control animals (15–18). Off-target genome editing at unintended sites, extensively studied *in vitro* (19–22) and *in vivo* (23–26), poses one of the most significant challenges in both basic research and translational applications of gene editing (27,28).

This rising number of gene-edited animals being generated is accompanied by an escalating number of off-target events (15–18,29). The analysis of off-target effects is essential for phenotype analyses, safety assessments and the practical application of these gene-edited animals. From a practical perspective, there is an urgent need to understand the biological phenomena associated with variations caused by off-target gene editing, such as genomic locations, variation types (e.g. single-nucleotide variants [SNVs] or small insertions and deletions [INDELs]) and allele frequencies, and to assess their subsequent effects on phenotype and health. However, this information is scattered through different studies and various databases, making it difficult to gain clear insights into the off-target effects in these gene-edited animals. Towards an increasing understanding of the causes and mechanisms behind off-target effects (26,30,31), a variety of analytical methodologies have been employed to detect off-target events in such animals (15–18,29,32–36). Some studies have focused on examining algorithm-predicted off-target events in these animals (29,32) or comparing differences in genome variants between gene-edited and wild-type animals (29,33) without eliminating genetic variants inherited from parents. Other studies have identified whole-genome *de novo* variations based on deep sequencing data analysis of parent-offspring trios (8,34,35), which distinguish *de novo* variations from genetic background in these animals. Different analytical methods yield diverse results, which further increase the difficulty in achieving a comprehensive understanding of off-target effects. So, it is necessary to adopt unified and standardized methods for identifying genomic variations across different gene-edited animals. Deep whole-genome sequencing (WGS) data, especially when combined with parent-offspring trio analyses, provides a straightforward, universally applicable and more accurate approach to identifying whole-genome *de novo* variations in gene-edited animals.

Compared with a substantial increase in the generation of gene-edited animals and related omics data, integrated data resources and analyses for gene-editing associated variations remain scarce. A number of public variation databases have been established, such as dbSNP (37) and dbVar (38), the two major resources for archiving genome variations for humans; ClinVar (39), a public archive of human variations classified for diseases and drug responses; GVM (40,41) as a public repository of genome variations for a range of species and iDog (42) as an integrated resource recording variations for domestic and wild canids.

Most current variation databases focus on variations in humans or naturally born animals, with a notable absence of databases that focus on variations and their functional impacts in gene-edited animals. It is imperative to establish a publicly accessible platform for archiving, analysing and presenting genomic variations and their impacts in these animals. This process includes the collation of WGS data of gene-edited animals, the identification of whole-genome variations using standardized methods, the assessment of their functional impacts and the enabling of data sharing.

Here, we present a database named Variation Database of Gene-Edited animals (VDGE, https://ngdc.cncb.ac.cn/vdge), an open-access repository that curates and integrates genomic variation and annotation data of multiple gene-edited animal species. Currently, VDGE houses off-targeting analyses conducted through unified and standardized workflows for multiple animal species, including monkeys, pigs, dogs and mice, encompassing 151 on-target mutations from 210 samples, and 115,710 variations identified from 107 gene-edited and wild-type animal trios, with comprehensive annotation details for each variation. It provides a free, one-stop service for researchers involved in gene-edited animal studies for browsing, searching, visualizing and downloading information on variations.

## Materials and methods

### Data collection

The WGS data of gene-edited and wild-type animals were collected from published literature or generated by this group. The criteria used in the selection of data from literature published before 1 May 2024 included gene-edited or wild-type monkeys, pigs, dogs and mice and their parents with WGS data and the coverage of WGS data had to exceed 20×. A total of 70 WGS samples of monkeys, 65 WGS samples of dogs and 39 WGS samples of mice that met the aforementioned criteria were selected for further data processing as shown in Table 1 (8,15,17,34,43–46). These 174 WGS samples were derived from 107 animal trios, where each trio was composed of three samples: one individual with its two parents. Raw sequence data were downloaded from the Sequence Read Archive (SRA) (47) and the Genome Sequence Archive (GSA) (48), as seen in Table 1.

**Table 1.** Data summary of the VDGE database

*Species*	Gene editors	Animal trios	Samples	On-target mutations	SNVs	INDELs	Genes	WGS data source
*Macaca mulatta*	SpCas9	7	10	3	413	150	226	NCBI SRA
	NA	8	18	0	163	109	153	NCBI SRA
*Macaca fascicularis*	SpCas9	1	3	1	36	7	24	NCBI SRA
	BE4max	27	33	14	107,452	357	9,182	NGDC GSA
	NA	4	6	0	181	320	319	NGDC GSA
*Sus scrofa*	ZFN	0	4	4	0	0	1	-
	TALEN	0	2	4	0	0	1	-
	SpCas9	0	5	7	0	0	1	-
	BE3	0	5	41	0	0	3	-
*Canis lupus familiaris*	SpCas9	17	28	23	481	2,121	1,326	NGDC GSA
	NA	14	39	0	399	249	362	NGDC GSA
*Mus musculus*	SpCas9	7	20	31	201	90	124	NCBI SRA
	BE3	0	7	13	0	0	0	-
	BE4	9	9	10	1,925	193	988	NCBI SRA
	NA	13	21	0	578	285	395	NCBI SRA

Note: GSA, Genome Sequence Archive; INDEL, small insertion and deletion; NCBI, National Center for Biotechnology Information; NGDC, National Genomics Data Center, Beijing Institute of Genomics, Chinese Academy of Sciences; SRA, Sequence Read Archive; SNV, single-nucleotide variant; TALEN, transcription activator-like effector nuclease; WGS, whole-genome sequencing; ZFN, zinc finger nucleases; NA, not applicable as they are wild-type animals; -, no WGS data available.

In addition to the gene-edited animals with trio data available, there is a vast array of those lacking trio information. Most related studies only present data on the on-target sites. We have collated a small number of these studies into the current database version, including 36 samples and 95 on-target mutations (49–58), as seen in Table 1. One such on-target gene is *myostatin* (*MSTN*) gene in pigs and dogs. MSTN is a negative regulator of skeletal muscle mass, which is closely related to the meat yield of agricultural animals (49,53).

The reference genomes used in this study included the rhesus monkey (*Macaca mulatta*: Mmul_10, GCF_003339765.1) (59), pig (*Sus scrofa*: Sscrofa11.1, GCF_000003025.6) (60), crab-eating macaque (*Macaca fascicularis*: MFA1912RKSv2, GCF_012559485.2) (61), dog (*Canis lupus familiaris*: Dog10K_Boxer_Tasha, GCF_000002285.5) (62) and mouse (*Mus musculus:* GRCm39, GCF_000001635.27) (63). Reference genomes and corresponding annotation feature files were downloaded from the NCBI Genome (https://www.ncbi.nlm.nih.gov/datasets/genome) (64). Gene Ontology (GO) information of various species was downloaded from AmiGO 2 (https://amigo.geneontology.org/amigo) (65).

### Data processing

Prior to detecting variations in gene-edited and wild-type animal trios, the genetic relationship between the offspring and their parents was first validated by analysing identity by descent (IBD) using PLINK1.9 software (66). Read sequences were then filtered using the fastp software (v 0.23.1) (67) with default parameters. The qualified short reads of all samples were mapped to reference genomes using the bwa-mem2 (v2.2.1) algorithm (68). Following the initial alignment, GATK (v4.2.6.1) (69) was used to sort the aligned BAM files and mark duplicate reads. Platypus (v0.8.1) (70) was employed to identify SNVs and INDELs from de-duplicated BAM files. We compared four variant calling tools, GATK (v 4.2.6.1) (69), Platypus (v0.8.1) (70), FreeBayes (v1.3.6) (71) and Strelka2 (v2.9.10) (72), and found that Platypus identified the highest number of known on-target mutations with a minimal occurrence of false positives compared to the other three tools. We, therefore, selected Platypus as the final variant calling tool.

The criteria for identifying *de novo* variations from variant call format (VCF) files of each animal trio called by Platypus were as follows: (i) The site filtering strategy filters variants at the site-level, taking variant quality by depth (QD), mapping quality (MQ), *P*-value for strand bias (SbPval), the number of forward reads (NF) and the number of reverse reads (NR) into consideration. (ii) The genotype quality (GQ) had to be no <40 for SNVs and no <5 for INDELs. (iii) For SNVs, the read depth was required to be between one-third and twice the average depth of each individual; for INDELs, the read depth was required to be between 5 and twice the average depth of each individual. (iv) The occurrence of *de novo* variations in the offspring was identified in accordance with the principles of Mendel’s genetic law. This means that the *de novo* variation in the offspring was heterozygous (0/1), whereas the genotype at the identical locus from both parents was homozygous (0/0 or 1/1). (v) The number of reads supporting the alternative allele of the offspring in the parents had to be no >1. (vi) The variant allele frequency defined as alternative allele/alternative allele + reference allele in the offspring had to exceed 0.2. (vii) Any missing genotype mutations present in a sample were excluded.

To remove false positive mutations from the filtered *de novo* variations, IGV (v2.16.1) (73) and the tview module of Samtools (v1.6) (74) were employed to examine the reads associated with all *de novo* variations. This process was conducted on the cleaned BAM files. A maximum of one read carrying an alternative allele at the variant position in the parental alignment BAM files was permitted. The term alternative allele read was defined as a read that aligned with the corresponding alternative allele observed in the offspring.

Finally, annotated information including genic and intergenic regions, variant effects, variant consequences and genes were obtained by comparing the variations against NCBI resources (64) using ANNOVAR (75). Gene symbols and names were standardized to vertebrate gene nomenclature committee (VGNC) nomenclature (76). Gene groups were delineated based on HUGO Gene Nomenclature Committee (HGNC) resources (77). The GO terms were identified through the GO knowledge base (78).

### Database implementation

The VDGE database was implemented using Spring Boot, a standalone Java application (https://spring.io/projects/spring-boot) as the backend framework. The frontend user interface was developed using Vue.js, an approachable and versatile framework for building web user interfaces (https://vuejs.org) and Quasar, an enterprise-ready cross-platform Vue.js framework (https://quasar.dev). All metadata were stored in MySQL, a free and popular relational database management system (https://www.mysql.com). For data visualization, JBrowse 2 (79) was used for secondary development. The charts on the web page were constructed using Apache ECharts, an open-source JavaScript visualization library (https://echarts.apache.org).

## Database content and usage

### Overview of VDGE

**Figure 1.**
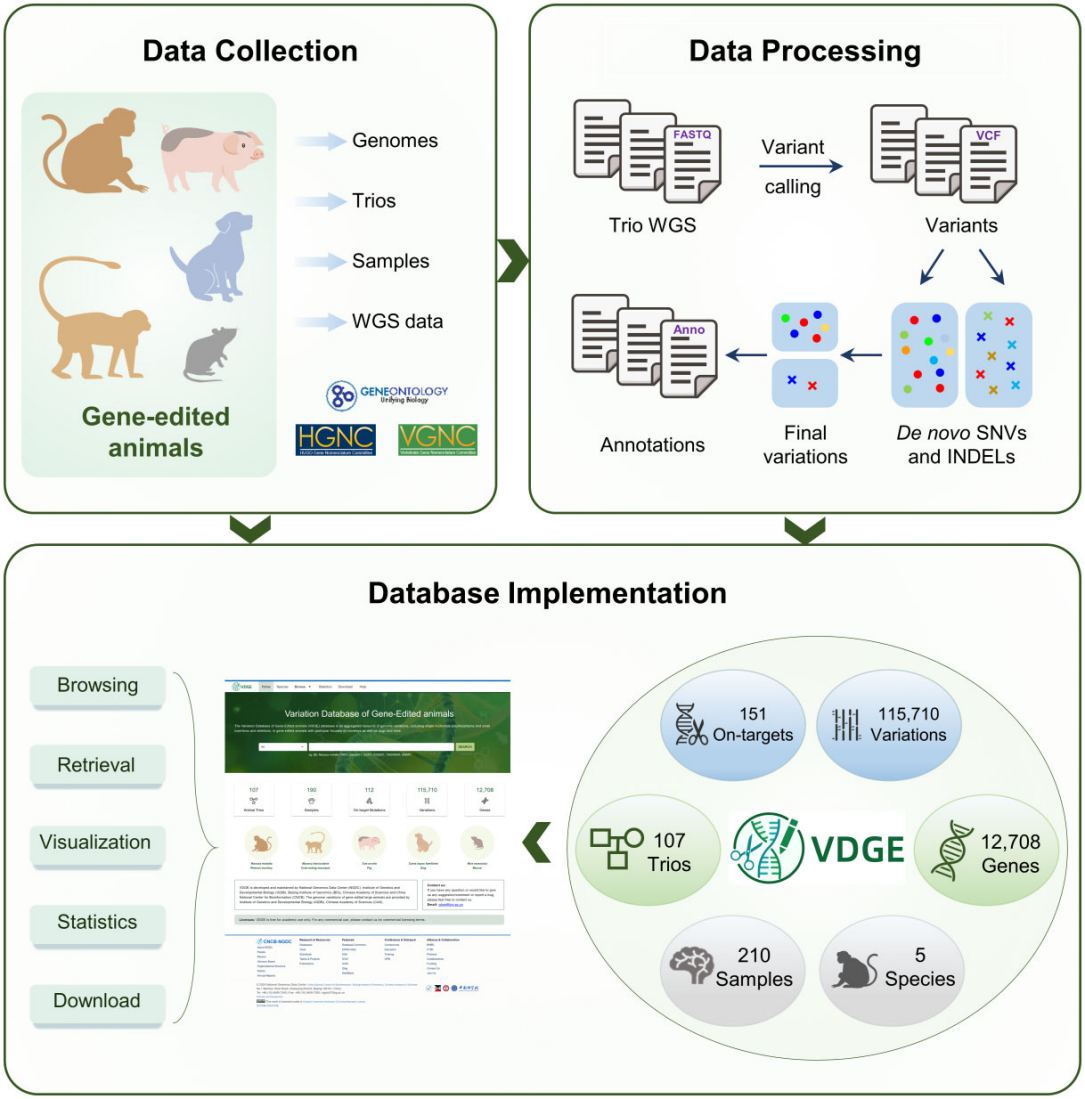
The construction pipeline of VDGE, including data collection, data processing and database implementation. INDEL, small insertion and deletion; SNV, single-nucleotide variant; WGS, whole-genome sequencing.

VDGE is a comprehensive platform that presents genomic variations and their potential functional impacts in gene-edited animals, offering users a one-stop solution for data acquisition and analysis across multiple species (Figure 1). It adopts an integrated architecture comprising six pivotal modules including Species, Animal Trios, Samples, On-target Mutations, Variations and Genes. The Variations and On-target Mutations modules interface with other modules to catalogue every variation identified by a standardized analytical pipeline in both gene-edited and wild-type animal trios (see ‘Materials and methods’ section). In the current version, VDGE presents a total of 115,710 variations and 56 on-target mutations, identified from 174 samples of 107 animal trios across four distinct species (8,15,17,34,43–46) using the standardized analysis pipeline. VDGE also includes 95 on-target mutations derived from 36 gene-edited animal samples which lack trio deep sequencing data. This information was collected and curated manually from publicly available literature (49–58). Additionally, 12,708 genes associated with variations were incorporated into the database. Data statistical information is summarized in Table 1.

### Species

**Figure 2.**
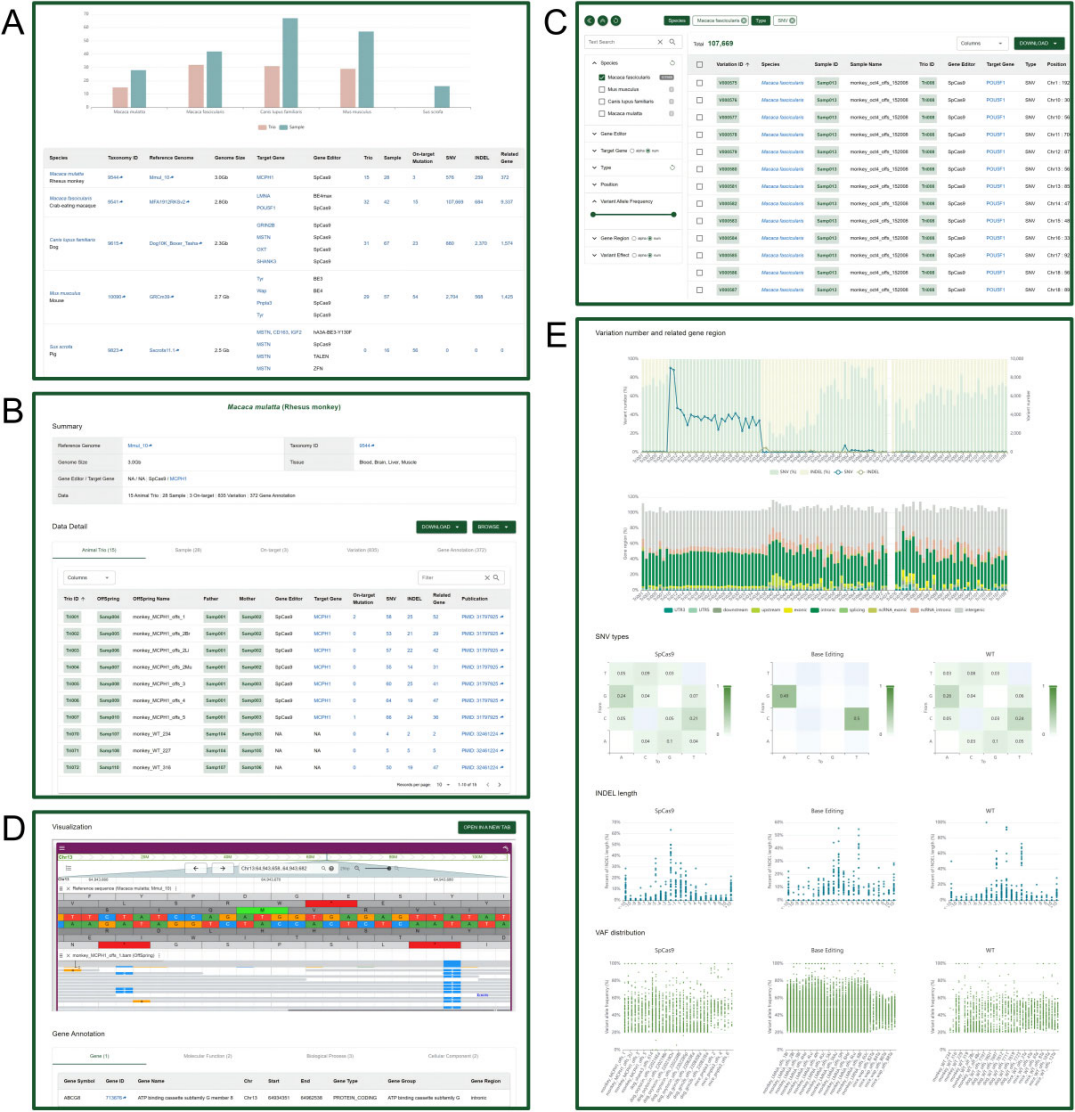
Screenshots of VDGE. (**A**) Data summary in the Species module. (**B**) Detailed page for the *Macaca mulatta* species. (**C**) Variations module. (**D**) Details of the variation V0000003, including JBrowse visualization and related gene ontology information. (**E**) Statistics module.

The Species module organizes diverse data spanning different species, providing users with a comprehensive overview of the contents of the database. VDGE currently contains data for gene-edited animals from four large animal species including the rhesus monkey (*M. mulatta*), the crab-eating macaque (*M. fascicularis*), the pig (*S. scrofa*) and the domestic dog (*Canis lupus familiaris*), as well as the house mouse (*M. musculu*s) as a classic animal model. The Species browsing page presents basic information for each species, including taxonomy ID, reference genome and genome size. It also incorporates concise statistical summaries of variations and related data including animal trios, samples, on-targets, variations and genes (Figure 2A), allowing users to navigate and access data resources for species of interest. For each species, VDGE offers a detailed page containing an overview and tabular displays of integrated data from other modules (Figure 2B).

### Animal Trios and Samples

The Animal Trios and Samples modules provide detailed information on trios and samples from both gene-edited and wild-type animals. Currently, VDGE houses 15 *M. mulatta* trios, 32 *M. fascicularis* trios, 31 *Canis lupus familiaris* trios and 29 *M. musculus* trios (Table 1). Each trio includes three samples from father, mother and their offspring (80). Animal Trios module provides comprehensive data sets, which are derived from other modules, including on-targets, variations and genes. These data sets are closely linked to the offspring samples. Each animal trio is assigned a unique identifier (ID) prefixed with ‘Tri’, while each WGS sample is tagged with ‘Samp’. These IDs serve as seamless navigational aids, enabling users to quickly access and explore data across diverse modules with ease and precision. The Animal Trios browsing page enables users to filter data of interest by species, gene editor and target gene, with the associated metadata presented in a tabular form. In addition, VDGE provides a detailed page for each animal trio, showing relevant information and tabular data from other modules. Similarly, the Samples browsing page permits the filtering of data based on sex, tissue, sequencing platform, sequencing depth and sequencing data access.

### On-target Mutations and Variations

The On-target Mutations module, designated with the prefix ‘On’, archives on-target variations induced by various gene editors in gene-edited animals. In parallel, the Variations module, characterized by unique identifiers commencing with ‘V’, stores variations including *de novo* SNVs and INDELs occurring at unintended genomic sites in the offspring samples of each gene-edited or wild-type animal trio. A total of 115,710 variations were identified though a standardized analysis pipeline from 107 animal trios (Table 1), with the majority of SNVs were derived from cytosine base editor (CBE)-edited animal trios. It is important to note that not all gene-edited animals exhibit on-target editing, as exemplified by cases such as Tri012 and Tri049, where the gene editing may not always occur at the intended on-target site (45).

Both the On-target Mutations and Variations browsing pages empower users to quickly filter data of interest based on a range of criteria in the left panel, including species, gene editors, target genes and variation types (Figure 2C). The metadata, presented in a structured table format, ensures clarity and accessibility. VDGE goes a step further by offering dedicated, comprehensive pages for each on-target mutation and variation. These pages are replete with metadata, encompassing detail information such as variation type, position, reference and alternative allele, variant allele frequency, variant effect, associated gene and phenotype. To enhance the user experience, JBrowse visualization and related gene ontology information are seamlessly integrated, providing a holistic view of the data (Figure 2D).

### Genes

To further evaluate the potential impacts of variations to gene-edited animals, each variation was annotated and subsequently mapped to the corresponding genes. All genes related to variations are included in the Genes module. A total of 12,708 genes were identified as being associated with all 115,710 variations in the database. Apparently, base editors (BEs) affect a much greater number of genes within an individual animal compared to *Streptococcus pyogenes* Cas9 (SpCas9) editors. The Genes page allows users to quickly filter data of interest based on species, gene symbol, position and gene type (e.g. coding or non-coding genes). By pinpointing the genes linked to variations, the impact of these genetic changes on the physiological status and health of gene-edited animals can be more readily evaluated.

### Data browsing, retrieval and download

VDGE provides data statistics and corresponding module entrances on the homepage. The search box allows users to input any keyword to search through all the data in the database or select the appropriate module to quickly explore data of interest. For each module, VDGE provides user-friendly browsing, filtering and retrieval interfaces. Users can filter data based on common entries. The resulting filtered data are presented in a clear, tabular format, and each module is interconnected through IDs, allowing users to navigate to any other data of interest. To further enhance users’ understanding, the Statistics module presents statistical metrics for select data, including variation number, gene region, SNV type, INDEL length and the distribution of variant allele frequency (Figure 2E). This statistical portrait facilitates the discovery of hidden data features.

To ensure data integrity for researchers to reuse, VDGE provides metadata and sequence data downloads. Users can click the Download button at the top right of each data table to download all metadata. For sequence data downloads, VDGE provides a separate Download module, which organizes all sequencing data, mapping data, SNV and INDEL data on a per-sample basis. Click on the link allows users to download the desired data from the provided FTP address.

### An example of using VDGE

The CBEs are a major class of BEs, which are engineered fusions of Cas9 and a cytidine deaminase enzyme that retain the ability to be programmed with a guide RNA and mediate the direct conversion of cytidine to uridine, effecting a C to T (or G to A) substitution (11). These CBEs have been reported to induce substantial genome-wide off-target SNVs in mouse embryos and rice (16,30).

**Figure 3.**
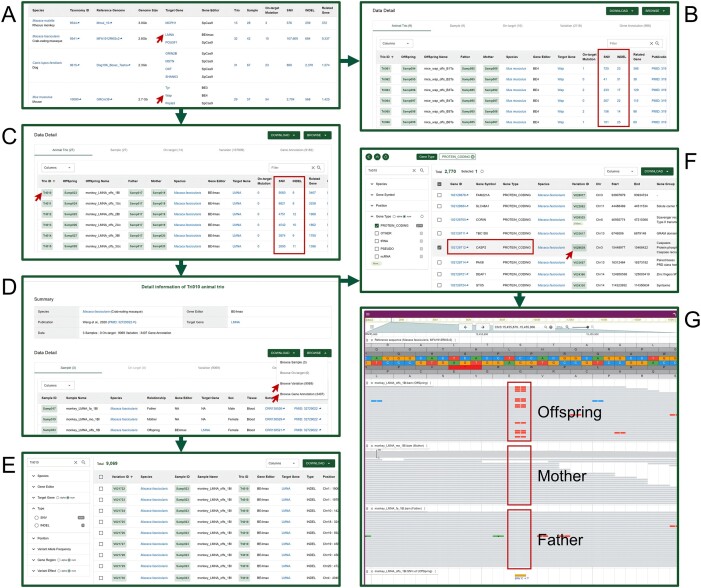
VDGE usage example. (**A**) Summarized panel for multiple species. (**B**) Mice trios edited by BE4. (**C**) Monkey trios edited by BE4max. The boxes indicate the number of SNVs and INDELs in each animal trio. (**D**) Detailed page for Tri010. Clicking on Browse Variation and Browse Gene Annotation buttons (indicated in D by arrows) will jump to Variations page (**E**) and annotated Genes page (**F**) of Tri010, respectively. (**G**) JBrowse visualization of *de novo* variation V028639 that is mapped to *caspase 2* (*CASP2*) gene (indicated by box). Clicking the buttons indicated by arrows in A, C, D and F will proceed to the subsequent page.

The information about BE-edited animal trios can be found in the Species module, by navigating to the Species button, which is prominently featured in the homepage’s navigation bar (Figure 3A). A fourth-generation base editor, BE4 (81), was used to target the *Wap* gene in *Mus musculus* (17). Ten on-targets and 2,118 variations were identified from nine mouse trios using the standardized analysis pipeline. These variations were mapped to 988 genes (Figure 3B). The number of SNVs in each trio was found to be in the hundreds, which is consistent with the findings of a previous study indicating that CBE treatment results in hundreds of genome-wide *de novo* SNVs in mouse embryos (16).

In parallel, BE4max, a variant of fourth-generation BEs (82), was employed to target the *LMNA* gene in *Macaca fascicularis* (45). Fourteen on-targets and 107,809 variations were identified from 27 monkey trios using the standardized analysis pipeline. These variations were mapped to 9,182 genes (Figure 3C). Navigating to the LMNA detail page and clicking on the Trio ID, information of a single trio can be viewed and monkey Tri010 was selected for further exploration. The detailed page for Tri010 displays the data source and information on the gene-edited animal and its parents. It shows that BE4max-mediated gene editing resulted in 9,069 d*e novo* variations in 3,407 genes in the gene-edited monkey. Related information for each sample can be viewed via external links (Figure 3D).

By clicking Browse button located in the upper right corner, the Variation browsing page is selected (Figure 3E). The filter bar on the left side of the page supports further filtering and viewing of the variations based on variation type, position, variant allele frequency, gene region and variant effect. Among the 9,069 d*e novo* variations, there are 9,060 SNVs and 9 INDELs, with the number of SNVs far exceeding that of INDELs (Figure 3E). On the Tri010 page, the Gene browsing page can be selected. The filter bar on the left side of the page allows for further filtering and viewing of the 3,407 genes related to the variations (Figure 3F). There are 2,770 protein-coding genes among all of the 3,407 genes. Variations in some of these genes may produce functional consequences for the gene-edited animals. For example, the variation V028639, which is mapped to the *caspase 2* (*CASP2*) gene, is a C to T point mutation (Figure 3G). The Variant Effect column shows that V028639 is a nonsynonymous SNV, resulting in the conversion of amino acids from glycine (G) to glutamic acid (E), as displayed in the Consequence column of the Variations module. Caspase 2 is a member of the caspases protein family, which is involved in cell death mediated by apoptosis, pyroptosis, necroptosis and autophagy (83). The functional disruption of Caspase 2 may affect the physiological status and health of the gene-edited animal (the offspring of the Tri010).

## Discussion and future plans

As a revolutionary biotechnology, gene editing holds immense promise and generates great expectations for its potential to transform various fields of biological research and application. Many gene editing therapies are currently in clinical trials and the FDA has approved a CRISPR-based therapy called Casgevy to treat sickle cell disease and β-thalassemia (84,85). Gene-edited animals are also the subject of a variety of fundamental biological studies and potential commercial applications (4,86–88).

The safety of gene editing is a significant concern for both researchers and the public (89). Off-target events in gene editing are rare in some cases (34,35) but not in others (16,30). Different gene editors produce varying off-target effects in the same cell line and the same gene editor can exhibit different off-target effects in different cell types (26). Even an identical gene-editing system can lead to different off-target events in different individuals. So, it is necessary to conduct a detailed off-target analysis for each individual gene-edited animal. In this study, we focused on the analysis of *de novo* variations in gene-edited animals with trio WGS data. It should be noted that not all *de novo* variations are caused by off-target effects of gene editing; some of them could be spontaneous mutations, including *de novo* germline mutations and somatic mutations (90).

The VDGE database is the first repository to present genome variations and annotations in gene-edited animals, with a particular focus on larger animals that hold a great application value. VDGE exhibits the following key characteristics: (i) VDGE offers a user-friendly platform that facilitates the exploration of genomic variation and annotation information across multiple gene-edited animal species, with efficient data browsing, retrieval and downloading capabilities, making it a one-stop resource for researchers seeking information on variations and offering a valuable dataset for the study of gene-edited animals. (ii) VDGE houses complete genomic variations for each animal trio by implementing a standardized analysis pipeline that leverages deep WGS data and parent-offspring trio analysis. (iii) VDGE provides an extensive dataset of variation-related information by integrating species, animal trios and on-target mutations, as well as annotation details such as variant type, genomic position, alternative allele and variant allele frequency, gene, gene ontology and potential functional consequence. These integrated data facilitate in-depth phenotype analysis, safety evaluations and translational studies for gene-edited animals. We recommend users to choose a computer browser to ensure the best user experience, even though our website is also compatible with mobile browsers.

In the future, VDGE will be maintained and updated by curating and integrating more on-target and variation information from gene-edited animals, particularly those lacking trio deep sequencing data. The range of organisms will be broadened by incorporating livestock, including cattle (86), sheep (91) and rabbits (92), as well as model organisms such as zebrafish (93), to meet the diverse needs of agricultural applications and scientific research. We also plan to enrich VDGE by incorporating variations identified by additional methodologies such as Cas-OFFinder (29), GUIDE-seq (15,23) and SITE-seq (18,21) to ensure a more comprehensive and accurate representation of variations present in each gene-edited animal. Moreover, a web server would be set up for the analysis pipeline used in this study. Once the new trio WGS data of gene-edited animals have been generated, the web server would facilitate the identification of variations. Furthermore, an upload functionality will be added to VDGE to allow users to submit on-target or variation information directly. A standardized data processing procedure will be implemented to ensure the consistency and accuracy of the data submitted by users and curated by VDGE staff.

## Data Availability

VDGE is available online for free at https://ngdc.cncb.ac.cn/vdge.
